# Fibroblast paracrine TNF-α signaling elevates integrin A5 expression in idiopathic pulmonary fibrosis (IPF)

**DOI:** 10.1186/s12931-017-0606-x

**Published:** 2017-06-19

**Authors:** Gali Epstein Shochet, Elizabetha Brook, Lilach Israeli-Shani, Evgeny Edelstein, David Shitrit

**Affiliations:** 1 Pulmonary Department, Meir Medical Center, 59 Tchernichovsky St, Kfar Saba, 44281 Israel; 2 Sackler Faculty of Medicine, Tel Aviv University, Tel Aviv, Israel; 3 Pathology Department, Meir Medical Center, 59 Tchernichovsky St, Kfar Saba, 44281 Israel

**Keywords:** Idiopathic pulmonary fibrosis (IPF), Fibroblasts, Integrin α5, TNF-α

## Abstract

**Background:**

Idiopathic pulmonary fibrosis (IPF) is a progressive lung disease with a poor prognosis. Inflammatory cytokines play a significant role in IPF pathology. However, the fibroblast itself is also believed to be the primary effector in IPF. We hypothesized that the fibroblasts themselves secrete pro-inflammatory cytokines that could propagate IPF by affecting normal neighboring cells. Thus, we explored the effects of IPF fibroblast derived media on normal fibroblast characteristics.

**Methods:**

Primary IPF/normal tissue derived fibroblast cultures were established and their supernatants were collected (IPF/N-SN, respectively). These supernatants were added to normal fibroblasts. Cell death (caspase-3, western blot), proliferation, viability (WST-1), migration (scratch test) and cell detachment (crystal violet and fibronectin adhesion assays) were tested. 10 inflammatory cytokines were measured by ELISA-based quantitative array. Integrin α5 (ITGA5), pIκBα, p/total STAT3 levels were measured by western blot/IHC. TNF-α involvement was confirmed using Infliximab ®, anti-TNF-α mAb.

**Results:**

The IPF-SN facilitated fibroblast cell detachment and reduced cell migration (*p* < 0.05). Nevertheless, these effects were reversed when cells were seeded on fibronectin. The exposure to the IPF-SN also elevated ITGA5 levels, the fibronectin receptor, in addition to NFκB pathway activation (pIκBα↑ 150%, *p* < 0.05). In accordance, IPF derived fibroblasts were found to express higher ITGA5 than the normal cells (44%↑, *p* < 0.05). ITGA5 was also expressed in the fibroblastic foci.

The IPF-SN contained high TNF-α levels (3-fold, *p* < 0.05), and Infliximab pretreatment successfully reversed all the above observations.

**Conclusion:**

We suggest a possible mechanism in which IPF fibroblast secreted TNF-α modifies neighboring fibroblast cell behavior.

## Background

Idiopathic pulmonary fibrosis (IPF) is defined as a specific form of chronic, progressive fibrosing interstitial pneumonia of unknown cause, occurring primarily in older adults, and limited to the lungs. It is characterized by progressive worsening of lung function, and is associated with a poor prognosis [1].

Although IPF is, by definition, a disease of unknown etiology, a number of potential mechanisms have been suggested over the years. Most patients with usual interstitial pneumonia (UIP), the pathologic hallmark of IPF, will manifest a mild to moderate degree of chronic cellular inflammation [1–3]. Therefore, the inflammation hypothesis has dominated the field of pulmonary fibrosis for decades, and IPF continues to be viewed by many authorities as a chronic inflammatory disease of the lung parenchyma [4, 5]. Like in other fibrotic diseases, IPF lungs have a persistent inflammatory stimulus that sustains and/or stimulates production of growth factors and fibrogenic cytokines, which together stimulate the deposition of connective-tissue elements that progressively remodel and destroy normal tissue architecture [6]. However, while an unremitting immune response can lead to fibrosis, immune-suppressive therapy affords limited benefits [1, 7]. Therefore, the hypothesis has been put forward that active cellular lung inflammation is not a major feature or a requirement for the development of IPF. Instead, the wide spectrum of proinflammatory and profibrogenic factors found in the IPF lung is considered to be a result of deregulated wound healing processes [8–10].

It is widely accepted that the microenvironment plays a significant role in disease progression [11]. Studies showed that fibrotic fibroblasts manifest pathological control of pathways governing cell proliferation, viability, motility, contractile function and connective tissue production [12, 13]. The increased deposition of matrix proteins seen in IPF may result from chronic stimulation of fibroblasts by lung effector cell derived cytokines, chemokines, and growth factors. Alternatively, similar signals may act on the existing heterogenous fibroblast population to mediate the emergence, whether by selection or induction, of sub populations of cells resulting in the predominance of the fibrotic phenotype in the lung [14], a phenotype that appears to remain in vitro [13].

Thus, on the one hand there is a wide consensus that inflammatory cytokines play a significant role in IPF progression, and on the other it is believed that the fibroblast itself is the primary effector in this disease progression. This can be settled by the fact that the fibroblasts themselves secrete pro-inflammatory cytokines, creating a pro-inflammatory microenvironment that further propagates IPF progression by affecting normal neighboring fibroblast cells. To test this hypothesis, we explored the effects of IPF derived soluble factors on normal fibroblast cell characteristics.

## Methods

### Fibroblast culture

Primary human fibroblasts were isolated by the explant culture method from 7 IPF (histologically confirmed) and 23 normal control tissue samples (histologically normal lung distant from a resected tumor) obtained at the time of biopsy. The ‘IPF group’ consisted of 57% males with age average of 62.5 ± 5 years, while the ‘Normal group’ consisted of 69% males with age average 67.1 ± 13 years.

The isolation was done as previously described [15]. Briefly, tissue samples were minced with sterile scissors and placed in a cell culture dish containing growth media. Over time, the fibroblasts migrated out of the tissue onto the surface of the dish. Excess tissue was removed. Following extraction, the fibroblasts were cultured in DMEM supplemented with 10% FCS, L-glutamine (2 mM), and antibiotics (Biological Industries, Israel). Cells were maintained in 5% CO_2_ at 37 °C. Cells designated fibroblasts in both IPF and controls had typical spindle morphology, were vimentin positive and cytokeratin negative. Primary fibroblasts were used up to passage 8. The cells were subcultivated twice weekly at a 1:3 split ratio, while the culture media were collected from the confluent culture flasks, centrifuged and stored at −80 °C for later use. Media was collected several times from each cell line (at each passage of the confluent flasks) up to passage 8 and as long as the cells proliferated normally.

### Exposing fibroblasts to supernatants

Fibroblast cells (2 × 10^5^) were placed in 24-well plates with/without fibronectin (FN) coating (R&D, 10 μg/ml) and allowed to adhere for 24 h prior to the beginning of experiments. Then, a scratch was made by a pipette tip, and 400 μl media from normal/IPF fibroblasts (as described above) was added for further culture. Notably, the supernatants for each experiment were taken from cell cultures of different patients. In addition, different patient derived primary normal fibroblasts were seeded for every biological repetition, thus achieving several combinations between the samples. The downside of this system is high variability in the effects and therefore, all results were normalized to the controls (in the same experiment). The n number refers to the total number of combinations used.

### Materials

Anti- tumor necrosis factor alpha (TNF-α) mAb, Infliximab (Remicade, Janssen Biologics, The Netherlands, kindly provided by Dr. Liron Berkovich) was diluted in PBS and added to the SN a few minutes before their addition to the cells.

### Cell count

Cell number and viability were evaluated by the NucleoCounter NC-200 Automated Cell Counter (ChemoMetec, Denmark). Results were verified by a manual count following a Trypan blue staining (Biological Industries, Israel).

### Viability and proliferation (WST-1) assay

Assessment of viability was performed using cell proliferation reagent WST-1 (Sigma, USA) according to manufacturer’s instructions.

### Assessment of apoptosis/necrosis

Annexin V-FITC supplemented with 1 μg PI (MEBCYTO® Apoptosis kit, MBL) served for detection of cell death by flow cytometry as previously described [16].

### Detachment assay

Following culture, cells were fixed in 4% paraformaldehyde (Sigma, USA) for 10 min, washed in PBS and stained with crystal violet (0.5%). Excess stain was washed with water. Crystal violet was solubilized in 1% SDS (Biological industries, Israel) and absorbance was measured at 595 nm.

### Cell migration (Scratch test)

Fibroblast cells (5 × 10^4^) were placed in 96-well plates with/without FN coating (10 μg/ml) and allowed to adhere for 24 h. Following wounding of the confluent cell layers, normal/IPF derived supernatants with/without treatment (100 μl) were added to the cells. Wound closure was monitored immediately after scratching (time 0) and at 24 h post wounding. Areas were measured using the ImageJ software.

### Fibronectin (FN) adhesion assay

Ninety six-well culture plates were coated with FN (R&D, 10 μg/ml) overnight at 4 °C. At the end of the experiment, part of the harvested cells (5 × 10^4^) from each treatment were seeded to these plates and allowed to adhere for 60 min at 37 °C. Following several washes, bound cells were counted.

### Western blotting

Fibroblasts/10 mg tissue samples were lysed and western blot was preformed as previously described [17]. For integrin A5 expression levels, proteins were extracted directly from the tissue sample (10 mg, following biopsy) and from the cell line culture flasks during regular cell passages (between passages 3–8, at the normal proliferating phase).

The following rabbit/mouse anti-human antibodies were used: proliferating cell nuclear antigen (PCNA) (Sc-7907), pIκBα (Santa Cruz biotechnology, USA, Sc-8404), caspase-3 (#9665), cleaved caspase-3 (#9664), Phospho - signal transducer and activator of transcription 3 (pSTAT3 Tyr-705, #9145), pSTAT3-ser727 (#9134), STAT3 (Cell Signaling Technologies, USA, #9139), GAPDH (ab9484), ITGA5 (Abcam, USA, EPR7854) and alpha-Tubulin (Sigma, USA, T5168). Bound antibodies were visualized using Goat peroxidase-conjugated secondary antibodies (Millipore, USA, anti-Mouse IgG #AP308P and anti-Rabbit IgG #AP307P) followed by enhanced chemiluminescence detection (Millipore, USA). Optical densities were visualized and measured as arbitrary units by LAS3000 Imager (Fugifilm, USA). Results were normalized to Tubulin and GAPDH using Multi-gauge V3.0 program (Fugifilm, USA).

### Immunohistochemistry (IHC)

Paraffin sections were deparaffinized, rinsed in PBS and immersed in EDTA buffer (pH = 8). Samples were blocked using goat serum (Sigma Aldrich) and incubated with anti-ITGA5 antibody overnight. Following washing, slides were incubated with horseradish peroxidase labeled polymer (Zytomed systems GmbH, Germany), washed, and developed with AEC chromogen (ScyTec Laboratories, Logan UT, USA). Isotype-matched control excluded non-specific staining.

### ELISA based antibody array

Inflammatory factors were measured using Quantibody Human Inflammation Array Q1 kit (RayBiotec, Inc., USA), according to manufacturer’s instructions.

### RNA extraction and RT cDNA synthesis

Fibroblasts were washed with PBS and RNA was extracted using the RNeasy kit (QAIGEN, USA). RNA concentrations were measured at 260/280 nm using Nanodrop-1000 (Thermo Scientific, USA). Extracted RNA was converted to cDNA using GeneAmp RNA PCR (Applied Biosystems, USA) according to manufacturer’s instructions.

### Real time quantitative PCR

Reactions were done using Power SYBR Green (Applied Biosystems, UK). The following primers were used (5′–3′): GAPDH: forward: CTCTGCTCCTCCTGTTCGAC; reverse: TTAAAAGCAGCCCTGGTGAC; ACTA2 forward: TGAGAAGAGTTACGAGTTGCCTGAT, reverse: GCAGACTCCATCCCGATGAA; COL1A forward: CGAAGACATCCCACCAATCAC, reverse: CAGATCACGTCATCGCACAAC; ITGA5 forward: AGGCCAGCCCTACATTATCA, reverse: GTCTTCTCCACAGTCCAGCA. GAPDH served as the housekeeping control. Primers were normalized by specific cDNA standard curves obtained from known amounts of cDNA (primers were purchased from Hylabs, Israel).

### Statistical analysis

Paired Student’s t-tests were employed to analyze differences between cohorts. An effect was considered significant when the *P*-value was <0.05. All experiments were conducted at least three times.

## Results

### IPF fibroblast derived supernatants moderately affect normal fibroblast survival

Normal tissue derived fibroblasts were exposed to normal (N-SN) and IPF fibroblast derived supernatants (IPF-SN) (see Methods section) and the fibroblast apoptotic/total cell death, viability, PCNA levels and cell number were tested. The N-SN served as control.

**Fig. 1:**
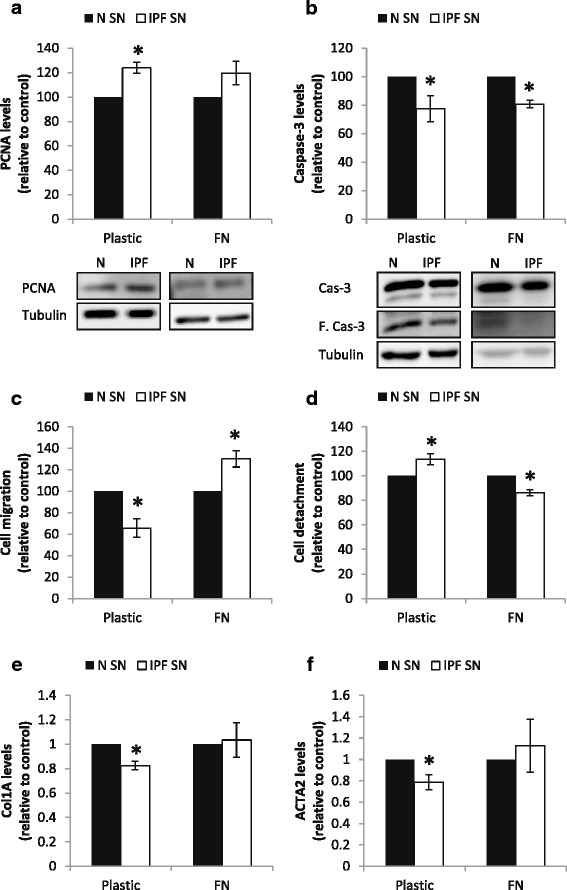
IPF fibroblast derived supernatants affect normal fibroblast migration and detachment in a fibronectin dependent manner. IPF/normal supernatants were added to fibroblast cells seeded on plastic or fibronectin (FN) (10 μg/ml) coated plates for 30 min (**a**-**b**) and 24 h (**e**-**f**). Following culture, cells were harvested for protein/RNA extraction and the effect of IPF derived supernatants (IPF-SN) on PCNA (**a**) and caspase-3 (**b**) levels were tested by western blotting (*n* = 4). The effect of the IPF-SN on fibroblast cell migration was tested by scratch test (**c**). Following the scratch test, cell detachment was quantified by crystal violet staining (**d**) (*n* = 9 and *n* = 4 for plastic and FN, respectively). Collagen 1a (COL1A) (**e**) and αSMA (ACTA2) (**f**) mRNA levels were tested by qPCR (*n* = 4). Results were normalized to control and considered significant * if *p* < 0.05, error bar represents the standard error

Following 24 h, the IPF-SN moderately, but significantly, elevated PCNA levels in the fibroblast cells following 30 min of culture (*p* < 0.05, Fig. 1a, no change at 24 h). This elevation was accompanied by an elevation in total cell number (50% elevation, *N* = 3, *p* < 0.01) but with no change in cell viability (measured using the WST-1 reagent and the automated cell counter). In addition, we found a reduction in total and cleaved Caspase-3 levels in these cells following 30 min (*p* < 0.05, Fig. 1b). However, these changes were relatively small and were not accompanied by changes in total or apoptotic cell death (measured by flow cytometry (no change, *N* = 4)). Overall, all the effects on cell death and proliferation were similar in cells cultured on plastic and on FN, suggesting that the FN had no influence on cell survival in this culture system.

### IPF fibroblast derived supernatants affect normal fibroblast migration and detachment in a fibronectin dependent manner

Normal tissue derived fibroblasts were exposed to the N-SN and the IPF-SN) and their cell migration was evaluated by scratch test. The N-SN served as control. Rather unexpectedly, the IPF-SN significantly reduced fibroblast cell migration (*p* < 0.01, Fig. 1c). This reduction was a result of both cell aggregations at the sides of the wound and of cell detachment from the plastic surface (*p* < 0.001, Fig. 1d). Moreover, the IPF-SN exposed cells displayed reduced levels of collagen 1a and alpha smooth muscle actin (αSMA) (ACTA2) mRNA levels (*p* < 0.01, Fig. 1e-f).

Nevertheless, these effects were reversed when the cells were seeded on FN prior to their exposure to the supernatants. Following exposure to the IPF-SN, the normal fibroblasts that were seeded on FN (10 μg/cm^2^) displayed increased migration and adhesion in comparison to cells exposed to the control supernatants (*p* < 0.05, Fig. 1c, d). Accordingly, the reduction in collagen 1a and αSMA levels was no longer observed (Fig. 1e-f); instead, the transcripts for both profibrotic genes indicated a slight (but insignificant) upregulation.

### IPF fibroblast derived supernatants elevate integrin α5 levels

**Fig. 2:**
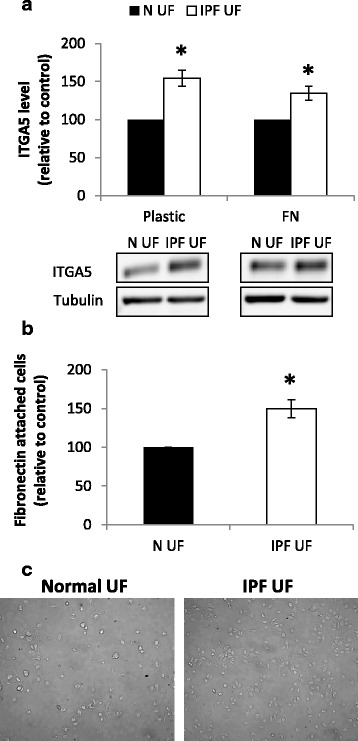
IPF fibroblast derived supernatants elevate integrin α5 (ITGA5) levels. IPF/normal supernatants were added to fibroblast cells seeded on plastic or fibronectin (FN) (10 μg/ml) coated plates. Following culture, cells were harvested for protein extraction and their adhesion to the FN was tested. Levels of ITGA5 were analyzed by western blotting (*n* = 13) (**a**). Cell adhesion to FN was quantified by microscopic cell count (*n* = 5) (**b**). Results were normalized to control (N-SN) and considered significant * if *p* < 0.05 (n ≥ 4), error bar represents the standard error. (**c**) Representative photomicrographs of increased FN attachment of fibroblast cells cultured with IPF-SN (*right*) following 24 h

Since the cell counts and proliferation marker were actually elevated following the culture with the IPF-SN, we considered the possibly that anoikis resistance mechanisms were activated. One of its markers is ITGA5 [18], which is also a part of the common FN receptor α5β1 [19]. Indeed, ITGA5 was significantly elevated following 24 h of exposure to the IPF-SN (*p* < 0.001, Fig. 2a). Accordingly, the culture with the IPF-SN also induced a significant elevation in cell adhesion to FN (*p* < 0.01, Fig. 2c).

Interestingly, fibroblasts that were seeded on FN prior to their exposure to the IPF-SN displayed a similar elevation in ITGA5 levels (*p* < 0.01, Fig. 2a, b). The ITGA5 mRNA levels were also tested and found to be slightly, but not significantly changed following 24 h (fold change of IPF-SN vs. control: 1.1 ± 0.07 and 0.94 ± 0.05 for plastic and FN, respectively, *N* = 7).

These results suggested that the trigger was present in the IPF-SN and was not only a result of cell detachment or an anoikis evasion mechanism.

### IPF fibroblasts express high levels of Integrin α5

**Fig. 3:**
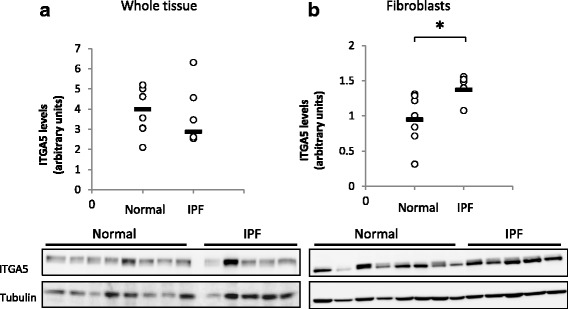
IPF derived fibroblasts express higher levels of Integrin a5 (ITGA5). ITGA5 levels were measured in whole tissue lysates (**a**) and IPF/normal derived fibroblast cells (**b**) by western blotting. (*n* = 7 and *n* = 6 for Normal and IPF, respectively). Results were considered significant * if *p* < 0.05

ITGA5 was shown to be elevated in lung cancer tissues, and was even suggested as a possible therapeutic target [20, 21]. Since IPF and cancer share a lot of common mechanisms, we tested whether IPF tissues and IPF derived fibroblasts expressed higher levels of ITGA5, in comparison to normal tissue samples. Although no difference was found between the IPF and healthy whole tissue lysates (Fig. 3a), the IPF derived fibroblasts expressed higher ITGA5 levels than the normal fibroblasts (*p* < 0.05, Fig. 3b).

**Fig. 4:**
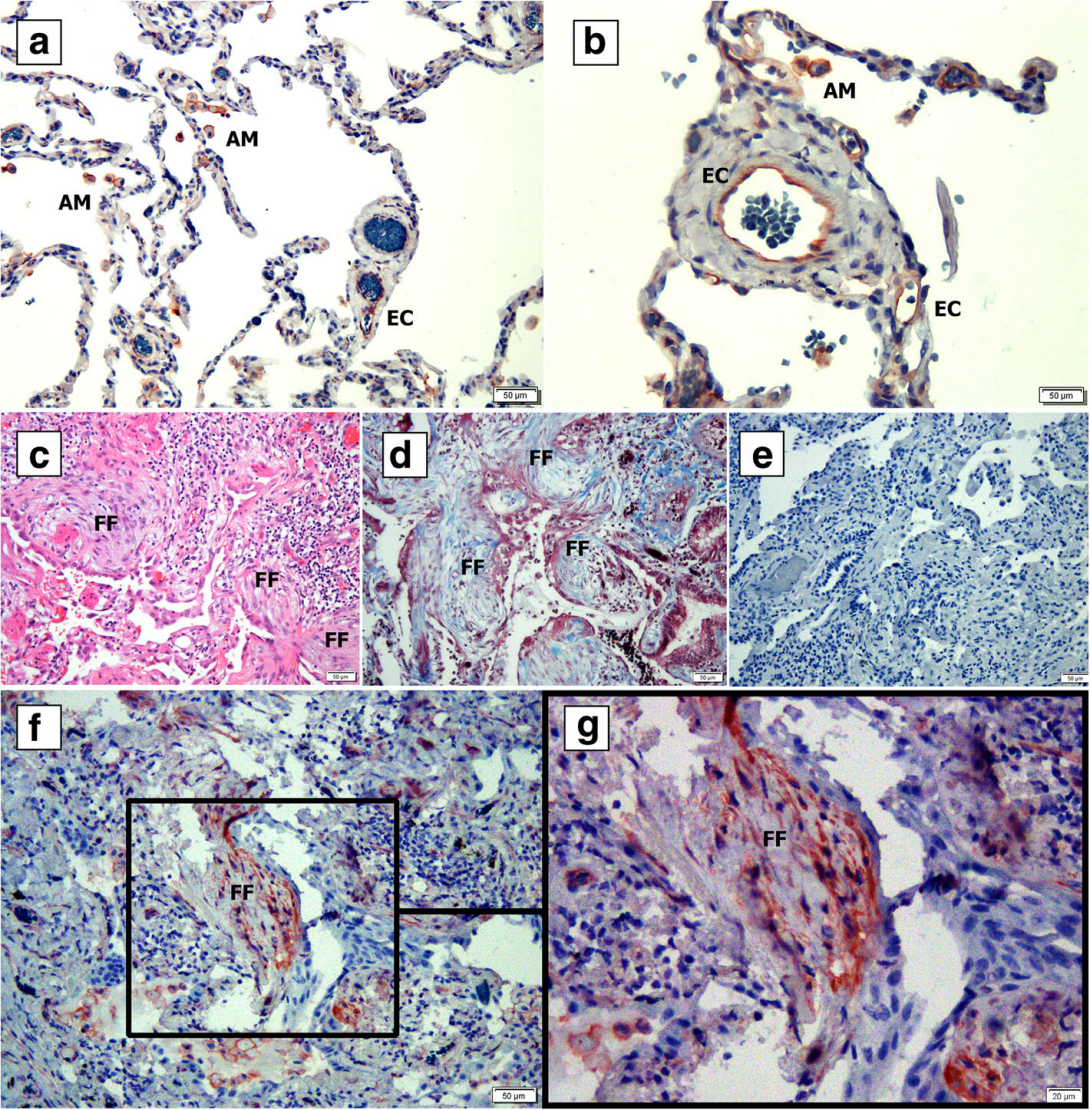
IPF fibroblastic foci express Integrin a5 (ITGA5). Normal (**a**-**b**) and IPF (**c**-**g**) lung tissue samples were fixed in formaldehyde and paraffin embedded. Following deparaffinization, slides were stained with hematoxylin and eosin (**c**), Masson-trichrome stain (**d**), IgG rabbit isotype control (**e**) and with anti-ITGA5 antibody (**a**-**b**, **f**-**g**). FF = fibroblastic foci; AM- Alveolar macrophages; EC = endothelial cells

In addition, we tested the localization of the ITGA5 in the normal and IPF tissue samples by IHC. We found that in addition to intra-alveolar macrophages (AM) and endothelial cells (EC) (Fig. 4a-b), the fibroblasts at the fibroblastic foci (FF) were positively stained to ITGA5 (Fig. 4f-g).

### Inflammatory factor expression in the fibroblast derived supernatants

Fibrosis is known to be linked to inflammatory responses (reviewed in [22]). Thus, we tested 10 inflammatory cytokine levels in the control and IPF fibroblast derived supernatants using ELISA based arrays. IL-1b, IL-4, IL-10 and IL-13 levels were nearly undetectable. Interestingly, the levels of TNF-α, IFN-γ and IL-8 were elevated by 2–3 fold in the IPF-SN in comparison to the control supernatants (*p* < 0.05, Table 1). These results show that not only immune cells, but the IPF derived fibroblasts themselves secrete increased amounts of inflammatory factors that could potentially affect normal cell behavior.

**Table 1:** pro-Inflammatory cytokine concentration in the culture supernatants

(pg/ml)	Normal SN (*n* = 9)	IPF-SN (*n* = 7)
IL-1a	0.4	2.7
IL-1b	1.4	0.0
IL-4	0.0	0.0
IL-6	2255 (±226)	2773.3 (±323)
IL-8	85.3 (±11)	215.8 (±26.4) *
IL-10	0.2	0.0
IL-13	3	0.0
MCP-1	772 (±96.7)	694 (±140)
IFN-γ	44.1 (±16.3)	136.1 (±58.1)
TNF-α	8 (±1.9)	15.7 (±2.1) *

* *p* < 0.05

### TNF-α mediates the ITGA5 elevation and induces NF-κB pathway activation

**Fig. 5:**
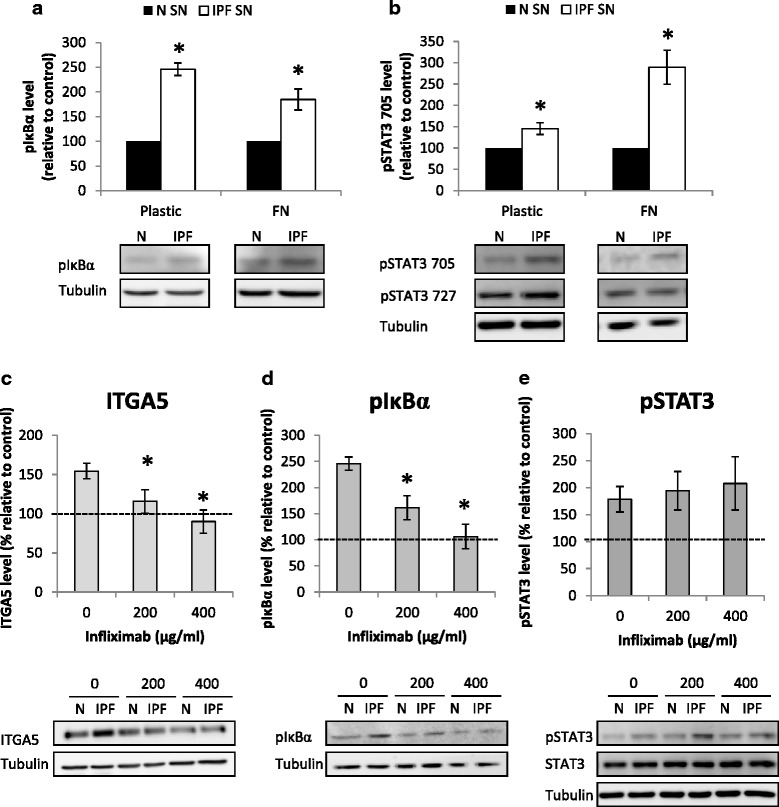
TNF-α mediates the ITGA5 elevation and NF-κB pathway activation. IPF/normal supernatants were added to fibroblast cells seeded on plastic or fibronectin (FN) (10 μg/ml) coated plates for 30 min. Following culture, cells were harvested for protein extraction and the effect of IPF derived supernatants (IPF-SN) on pIκBα (**a**) and pSTAT3- Tyr705 and pSTAT3- ser727 (**b**) levels were tested by western blotting (*n* = 4). Infliximab (200–400 μg/ml) was added to fibroblasts cultured with normal (N-SN)/IPF-SN. Levels of ITGA5 were analyzed by western blotting following 24 h (*n* = 8 and *n* = 4 for 200 and 400 μg/ml, respectively) (**c**) and of pIκBα (*n* = 4) (**d**) and pSTAT3-Tyr705 (*n* = 4) (**e**) following 30 min. Results were normalized to control (N-SN) and considered significant * if *p* < 0.05, error bar represents the standard error

Previous reports showed that the TNF-α could elevate ITGA5 expression via the NF-κB pathway in cancer [23]. We tested NF-κB pathway activation in the cells following 30 min of culture with/without IPF supernatants and found significant up-regulation of the pIκBα, both on plastic and on the FN coated plates (*p* < 0.05, Fig. 5a). STAT3 is another mediator of the inflammatory response (reviewed in [24]). We tested STAT3 phosphorylation (pSTAT) levels in these cells and found a significant elevation in the pSTAT3-Tyr705 on both surfaces as well (*p* < 0.05, Fig. 5b).

In order to validate TNF-α specific involvement, we added a TNF-α blocking antibody (200- 400 μg/ml, Infliximab) to the supernatants before their addition to the cells. The specific TNF-α blocker inhibited the elevation in ITGA5 and pIκBα levels in a dose dependant manner (*p* < 0.05, Fig. 5c, d). However, it had no effect on pSTAT3 levels (Fig. 5e). Thus we concluded that the TNF-α activates the NF-κB pathway and induces the ITGA5 elevation, and that the STAT3 pathway is probably activated by another cytokine excessively present in the IPF-SN, such as the IFN-γ.

### TNF-α blocking reverses the effects of the IPF secreted factors on cell migration and adhesion

**Fig. 6:**
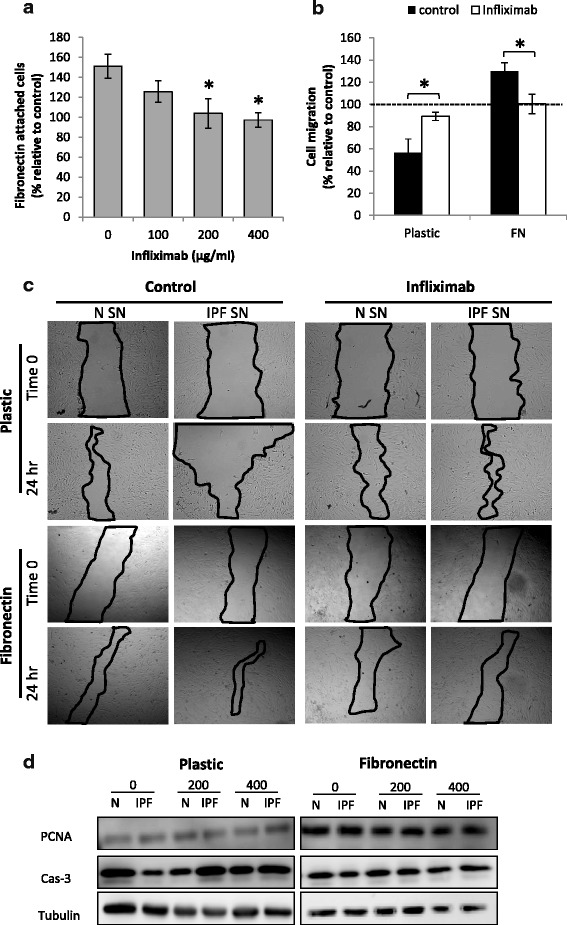
TNF-α blocking reverses the effects of the IPF secreted factors. Infliximab (100–400 μg/ml) was added to fibroblasts cultured with normal (N-SN)/IPF (IPF-SN) supernatants and the effect on cell adhesion to fibronectin (FN) was tested (*n* = 5) (**a**). IPF/normal supernatants with/without Infliximab (400 μg/ml) were added to fibroblast cells seeded on plastic or fibronectin (FN) (10 μg/ml) coated plates and cell migration was tested (*n* = 4) (**b**). Results were normalized to control (N-SN) and considered significant * if *p* < 0.05, error bar represents the standard error. Representative photomicrographs of the scratch test assays (**c**). IPF/normal supernatants were added to fibroblast cells seeded on plastic or FN (10 μg/ml) coated plates for 30 min. PCNA (*n* = 4) and caspase-3 levels were tested by western blotting. Representative figure of 4 independent experiments (**d**)

The elevation in ITGA5 was linked to increased cell attachment to FN. As a final step, we wished to test whether the TNF-α blocker could affect cell migration and cell attachment to FN. Thus, the cells were cultured for 24 h with the IPF/control supernatants with/without Infliximab (100–400 μg/ml), and their ability to attach to FN was tested. Notably, the Infliximab had no significant effect on the elevation in PCNA levels and cell numbers that were observed with the IPF-SN addition. Cell viability was not changed as well. However, the reduction in caspase-3 level was no longer observed (Fig. 6d). Indeed, the Infliximab completely prevented the elevation in cell attachment to FN that was induced by the IPF-SN (*p* < 0.05, Fig. 6a). In addition, it also prevented the changes in cell migration that were induced by the IPF-SN shown above in Fig. 1c (plastic and FN, *p* < 0.05, Fig. 6b). Moreover, the reduction in Collagen 1a and αSMA levels on the plastic surface (Fig. 1e-f) was also completely prevented (fold change of IPF-SN vs. control: 0.97 ± 0.05 and 1.06 ± 0.12 for COL1A and ACTA2, respectively, *n* = 4). On the FN, in correlation with the migration results (Fig. 6b), Infliximab addition resulted in a modest reduction in COL1A and ACTA2 levels (fold change of IPF-SN vs. control-FN: 0.78 ± 0.09 (*p* = 0.05) and 0.79 ± 0.17 (NS) for COL1A and ACTA2, respectively, *n* = 4).

These results show that the TNF-α, secreted from the IPF fibroblasts, induces cell migration and attachment to FN, which are probably a result of elevated ITGA5 levels.

## Discussion

Metastatic and fibrotic processes were shown to have a lot in common [22, 25]. The importance of the secretome (i.e., the secreted factors) to tumor progression has been established [26]. In this study, we tested the effect of the IPF fibroblast derived supernatants on normal fibroblast cell behavior. In fact, we found small, yet significant, differences between IPF and control supernatants on cell survival. These findings resemble our previous study, in which we compared normal and tumor derived supernatants effects on NSCLC survival [16]. Nonetheless, the IPF-SN had an inhibitory effect on cell migration and adhesion, which was reversed in cells cultured on FN. Indeed, Varner et al. [19] reported that cell behavior depends on the adhesion to FN and on ITGA5 subunit expression. Moreover, a recent study showed that the migration ability of dermal fibroblasts improved following their exposure to FN [27].

Apoptosis that results from cell detachment is termed anoikis. Since there was no elevation in cell death, we suspected that anoikis resistance mechanisms were activated in the detached cells. As previously reported in cancer, ITGα5 elevation [18] and activation of the NF-κB pathway [28] are part of these mechanisms. Thus, we tested ITGα5 and pIκBα levels in normal cells following their exposure to the IPF supernatants and found them to be significantly elevated. Nevertheless, in the cells that were seeded on FN that were no longer detached by the IPF-SN, ITGα5 and pIκBα levels were still elevated. This suggested that the up-regulation was not only a result of anoikis resistance, but triggered by a soluble component in the IPF derived media.

Following the above results, we concluded that the FN is essential in the fibroblast cell response to the IPF fibroblast secreted factors. Fibronectin is a large glycoprotein found in the plasma and extracellular matrices, which is secreted by fibroblasts [29] and a variety of other cells. For decades it is known that tissue samples and bronchopulmonary lavage (BAL) fluids of interstitial lung disease patients contain higher FN levels than normal tissues [30]. Several studies highlighted the central role of α5β1 integrin/FN interaction in the promotion of tumor progression [20, 21]. To establish a similar connection in IPF, we tested ITGA5 levels in whole tissue lysates and IPF/normal fibroblasts by western blotting. Although no difference was found between the IPF and control, we found that IPF derived fibroblasts expressed higher levels of ITGA5 in comparison to normal tissue derived cells. As previously suggested [13], these findings show that the IPF derived fibroblasts present a different phenotype that is maintained in vitro. IHC staining of IPF biopsy samples revealed that in addition to the fibroblasts, intra-alveolar macrophages and endothelial cells were also positively stained, suggesting an explanation to the lack of difference between tissue lysates. This finding warrants further study and suggests that targeting the ITGA5 locally may serve as a therapeutic approach.

Fibroblasts are known to secrete multiple growth factors and cytokines in culture. Similarly to ours and others previous studies [16, 26], we only used media that contained 10% FCS and avoided the use of serum-free media. In order to avoid the bias of the cytokines present in the FCS, similar FCS lots were used in the culture of the normal and the IPF cells.

Since there is a wide consensus that inflammatory cytokines play a significant role in IPF progression, we compared 10 inflammatory cytokine expression levels between the IPF and normal SNs. Indeed, we found that the levels of TNF-α, IFN-γ and IL-8 were higher in the IPF-SN in comparison to the normal fibroblast supernatant. Since IL-8 seems to be less involved in the pathogenesis of IPF than previously thought [31] and the involvement of IFN-γ in IPF was extensively studied yet failed to show significant benefit in several large clinical trials [1, 32], we decided to focus our research on TNF-α.

TNF-α is a pleiotropic cytokine expressed by many cell types in response to infection or injury. Inappropriate production of TNF has been implicated in the pathogenesis of a variety of human diseases, including sepsis, diabetes, cancer, osteoporosis, allograft rejection, multiple sclerosis and rheumatoid arthritis (RA) [33], including pulmonary fibrosis [8, 9, 34]. Although no differences were found between IPF patients and healthy controls in the serum levels of TNF-α [35], elevated levels of TNF-α were detected in the lungs of experimental pulmonary fibrosis animal models [36] and it was shown to be expressed locally in the lungs of IPF patients [8, 9]. These findings suggest that there could be a local production of TNF-α, possibly by the fibroblasts.

Recently, TNF-α was shown to affect multiple responses that extend well beyond its well characterized pro-inflammatory properties. For example, Verjee et al. showed that TNF selectively induces the myofibroblast phenotype in Dupuytren’s palmar fibroblasts, and that these effects could be reversed by anti-TNF antibodies [37]. Moreover, it was found that TNF-α upregulates leukocyte adhesion to FN during culture by increasing surface expression of α5β1 integrins [23]. In their study, Li B et al. concluded that the elevation in the ITGA5 subunit was mediated by the NF-kB pathway [23]. Indeed, we found significant activation of the pIκBα by the IPF-SN both on plastic and FN, suggesting it to be activated by a soluble factor.

STAT3 is a key transcription factor regulating human fibroblast-myofibroblast differentiation and homeostasis, and as such, likely plays a role in the pathogenesis of UIP/IPF [38]. STAT3 activity is primarily dependent on the level of phosphorylation at Tyr705, which is activated through tyrosine phosphorylation in response to factors such as the IL-6 and IFN-γ [39]. In our culture system, we found significant activation of STAT3 signaling, supporting previously published data regarding its relevance in pulmonary fibrosis [38].

As a final step, we selectively blocked TNF-α activity and showed that the elevation in ITGA5, pIκBα and the increased cell adhesion to FN and migration on FN all could be reversed. The pSTAT3 levels however were not affected, suggesting that the trigger for its activation was not TNF-α, but possibly the IFN-γ that was also elevated in the IPF supernatants. IPF fibroblasts are known to have increased migration ability, as well as elevated Collagen 1a and αSMA levels [12]. Throughout the study, the migration results following exposure to the IPF-SN correlated to the collagen 1a and aSMA transcript expression levels, further supporting our findings.

Infliximab (Remicade) is a chimeric monoclonal antibody that binds both soluble and membrane forms of TNF-α and neutralizes its biological effects [40]. In vitro studies on embryos showed no toxic effects at doses as high as 200 μg/ml [41]. Importantly, Infliximab is not known to bind to any antigen other than TNF [42]. Infliximab is currently approved for the treatment of RA and Crohn’s disease, but its benefit in IPF treatment was already suggested [43, 44]. Moreover, a recent study by Altintas et al. showed that Infliximab pretreatment can attenuate bleomycin induced pulmonary fibrosis in rats [45]. Nevertheless, Etanercept, a recombinant soluble human TNF receptor, treatment failed to show any significant effects in clinical trials for IPF [1, 46]. A comparison between Etanercept and Infliximab in Crohn’s disease outcomes showed different clinical results and the same can be assumed in pulmonary fibrosis. As reported by Scallon et al., Infliximab formed more stable complexes with TNF than the Etanercept, and that there was a more complete and sustained neutralization of TNF by the Infliximab [42].

## Conclusion

In this study we suggest a possible mechanism in which IPF fibroblast secreted TNF-α can modify neighboring fibroblast cell behavior. This soluble microenvironment induces the attachment of normal fibroblasts to FN via the elevation in ITGA5, which eventually can lead to focal accumulation of fibroblasts in the fibrotic fibronectin rich lung. Pretreatment with Infliximab reversed these observations. Although further in vivo models are warranted, our findings suggest the Infliximab as a possible therapeutic option for the treatment of pulmonary fibrosis in a localized manner.
